# PP86 Cost Effectiveness Of Maternal Respiratory Syncytial Virus (RSV) Prefusion F (RSVpreF) With Complementary Nirsevimab Versus Nirsevimab Alone For Preventing RSV Among Infants In Chile

**DOI:** 10.1017/S0266462325103036

**Published:** 2025-12-29

**Authors:** Rafael Bolaños, Juan Francisco Falconi, Ahuva Averin, Erin Quinn, Amy Law, Diana Mendes

## Abstract

**Introduction:**

RSV is a leading cause of lower respiratory tract illness (LRTI) among infants in Chile. Nirsevimab was recommended for infants in 2023 to address the burden of RSV-LRTI. The cost effectiveness of maternal RSVpreF with complementary nirsevimab for infants, versus nirsevimab alone, for prevention of RSV-LRTI among infants in Chile was evaluated.

**Methods:**

A cohort model to depict clinical and economic outcomes associated with RSV-LRTI for infants from birth to one year of age and lifetime consequences of RSV-related death. Results were based on disease and case-fatality rates, effectiveness, utility and disease-related disutility, and direct and indirect costs. The price of nirsevimab was USD260.00. The RSVpreF price was based on the economically justifiable value (EJV) of RSVpreF versus no intervention at a willingness-to-pay of one times the gross domestic product per capita. Uptake was set at 90 percent. In the complementary strategy, nirsevimab was limited to infants born to unvaccinated mothers. The healthcare system and societal perspectives were used, with costs in USD for 2023 and an annual discount rate of 3 percent.

**Results:**

With no intervention, 10,457 patients with RSV were hospitalized (RSV-H) and 39,460 attended the emergency department (RSV-ED) in the model, with corresponding total direct and indirect costs of USD21.0 million. Nirsevimab alone would prevent 52 percent (n=5,418) of RSV-H cases and 39 percent (n=15,317) of RSV-ED cases, at a total cost of USD58.8 million. Maternal RSVpreF with complementary nirsevimab would prevent 54 percent (n=5,635) of RSV-H and 34 percent (n=13,423) of RSV-ED cases. Using an EJV of USD94.34 per dose of RSVpreF, total costs of the complementary approach were lower by USD24.1 million, with an additional nine quality-adjusted life years gained. RSVpreF with complementary nirsevimab was cost saving, compared with nirsevimab alone.

**Conclusions:**

Use of RSVpreF with complementary nirsevimab would achieve a similar public health impact as nirsevimab alone and would yield substantial cost savings in Chile.

